# Physical exercise as a friend not a foe in acute kidney diseases through immune system modulation

**DOI:** 10.3389/fimmu.2023.1212163

**Published:** 2023-10-20

**Authors:** Ana Carolina Costanti-Nascimento, Leonilia Brelaz-Abreu, Elayne Bragança-Jardim, Welbert de Oliveira Pereira, Niels Olsen Saraiva Camara, Mariane Tami Amano

**Affiliations:** ^1^ Instituto Sírio-Libanês de Ensino e Pesquisa, Hospital Sírio-Libanês, São Paulo, Brazil; ^2^ Faculdade Israelita de Ciências da Saúde Albert Einstein, Hospital Israelita Albert Einstein, São Paulo, Brazil; ^3^ Departamento de Imunologia, Instituto de Ciências Biomédicas, Universidade de São Paulo, São Paulo, Brazil; ^4^ Departamento de Oncologia Clínica e Experimental, Escola Paulista de Medicina (UNIFESP), São Paulo, Brazil

**Keywords:** moderate exercise, immune system, cytokines, AKI -acute kidney injury, CKD - chronic kidney disease, regular exercise

## Abstract

Regular and moderate exercise is being used for therapeutic purposes in treating several diseases, including cancer, cardiovascular diseases, arthritis, and even chronic kidney diseases (CKDs). Conversely, extenuating physical exercise has long been pointed out as one of the sources of acute kidney injury (AKI) due to its severe impact on the body’s physiology. AKI development is associated with increased tubular necrosis, which initiates a cascade of inflammatory responses. The latter involves cytokine production, immune cell (macrophages, lymphocytes, and neutrophils, among others) activation, and increased oxidative stress. AKI can induce prolonged fibrosis stimulation, leading to CKD development. The need for therapeutic alternative treatments for AKI is still a relevant issue. In this context arises the question as to whether moderate, not extenuating, exercise could, on some level, prevent AKI. Several studies have shown that moderate exercise can help reduce tissue damage and increase the functional recovery of the kidneys after an acute injury. In particular, the immune system can be modulated by exercise, leading to a better recovery from different pathologies. In this review, we aimed to explore the role of exercise not as a trigger of AKI, but as a modulator of the inflammatory/immune system in the prevention or recovery from AKI in different scenarios. In AKI induced by ischemia and reperfusion, sepsis, diabetes, antibiotics, or chemotherapy, regular and/or moderate exercise could modulate the immune system toward a more regulatory immune response, presenting, in general, an anti-inflammatory profile. Exercise was shown to diminish oxidative stress, inflammatory markers (caspase-3, lactate dehydrogenase, and nitric oxide), inflammatory cytokines (interleukin (IL)-1b, IL-6, IL-8, and tumor necrosis factor-α (TNF-α)), modulate lymphocytes to an immune suppressive phenotype, and decrease tumor necrosis factor-β (TGF-β), a cytokine associated with fibrosis development. Thus, it creates an AKI recovery environment with less tissue damage, hypoxia, apoptosis, or fibrosis. In conclusion, the practice of regular moderate physical exercise has an impact on the immune system, favoring a regulatory and anti-inflammatory profile that prevents the occurrence of AKI and/or assists in the recovery from AKI. Moderate exercise should be considered for patients with AKI as a complementary therapy.

## Introduction

Acute kidney injury (AKI) is characterized by a sudden reduced glomerular filtration rate and, therefore, decreased renal function, with renal structural damage. In addition, AKI can lead to more severe diseases such as cardiovascular events and the development of chronic kidney disease (CKD) (1–4). AKI is common in all countries and has an increased risk of death at any stage. It occurs in 8%–32% of patients admitted to hospital and affects more than 50% of patients in intensive care units (ICUs) (1, 5). Although there is a high prevalence in ICU patients, AKI can also affect patients without a critical illness (6–8). The mechanisms that cause AKI are related to poor oxygen and nutrient delivery to the nephrons, an imbalance in reactive oxygen species (ROS) or nitric oxide (NO) production, and an increased energy demand. This leads to cell death through necrosis or apoptosis, or both, triggering a cascade of inflammatory response and renal damage and dysfunction (1, 9).

Physical exercise is a subcategory of physical activity that is planned and done with regularity to improve or maintain health and fitness (10–12). There are several ways to measure the intensity of exercise: training heart rate (THR), metabolic equivalent (MET—compares oxygen consumption during rest and during activity), and a certain percentage of the VO_2_max, which is the maximum oxygen consumption/uptake of an individual (11–13). Exercises are deemed to be extenuating if they cause each individual’s heart rate to reach 80% of its maximum level or if they cause them to consume ≥65% of their VO_2_ max over the entire exercise session (14, 15). Moderate exercise is typically defined as when an individual reaches 46%–63% of their VO_2_ max, attains a MET score of 3–5.9, or reaches 40%–60% of their THR (4, 13). With regard to the intensity of exercise, the studies included in this review followed those concepts of extenuating and moderate exercises, and most of them were based on the VO_2_ max percentage criteria. For exercise regularity, we used the same criteria as the American College of Sports Medicine Guidelines, in which exercises are considered regular when they are done three or more times a week for at least 4 weeks (10).

It is well known that extenuating exercise increases the risk of AKI (14–22). Strenuous exercise, in which study subjects had to run at their verified speed to elicit 65% VO_2_ max on a −10% gradient treadmill, led to dehydration and muscle damage. In addition, most of the time these exercises were carried out in a hot environment, and this combination appeared to exacerbate the risk of AKI (23). Exercise-induced AKI is characterized by muscle soreness, cramps or muscular stiffness, headaches, nausea, vomiting, and often changes in urine color. These symptoms are likely to be related to ischemic renal failure or kidney injury because of rhabdomyolysis (17, 24).

The immune system plays a major role in the pathophysiology of AKI and CKD (25, 26). In AKI, there is much necrotic cell death, damage-associated molecular patterns (DAMPs), monocytes, M1 macrophages, and proinflammatory cytokines, specifically interleukin (IL)-6, IL-8, and tumor necrosis factor-α (TNF-α) (26, 27). In addition, activated T cells contribute to nephrotoxicity, whereas B cells do not have a defined role in AKI pathogenesis and maintenance. This hyper-activated inflammatory response leads to more necrosis, which enhances inflammation, creating a vicious cycle of inflammation and renal tissue damage. In CKD, macrophages have an important role, producing IL-1β, TNF-α, and IL-6, which contribute to renal inflammation. Those cells also produce tumor necrosis factor-β (TGF-β) and promote extracellular matrix deposition, leading to renal fibrosis. T cells contribute to tubulointerstitial fibrosis by inducing tubular cell death and producing interferon-γ (IFN-γ) and TNF-α. B cells can have an important role in CKD pathogenesis depending on the cause of the disease; for example, if it is autoimmune, B cells can be the source of autoimmune antibodies that cause glomerulonephritis (25).

Physical exercise is closely related to our immune system. Exercise and muscle fatigue can cause the production of reactive oxygen species (ROS), induce adenosine triphosphate (ATP) depletion, and trigger an inflammatory reaction, leading to an increased number of T-lymphocytes after exercise (28). On the other hand, there is growing evidence that physical exercise also has an anti-inflammatory effect on the human body (4). Excess visceral adipose tissue causes a systemic inflammatory environment through the production of adipokines, including TNF-α and IL-6 (29, 30). In addition, obesity predisposes t-lymphocyte and macrophage infiltration in the visceral adipose tissue, which maintains the inflammatory state (29). Therefore, exercising acts as an anti-inflammatory stimulus by reducing the adipose tissue, and a randomized control trial showed that the bigger the adipose tissue loss, the greater the anti-inflammatory effect of exercise (31). That trial was done under a training protocol with aerobic exercises for 12 weeks, with intensities ranging from 50% to 85% VO_2_ max, and also showed that contracting skeletal muscles produces anti-inflammatory myokines, such as IL-6, IL-15, and irisin (31). IL-6 myokine results in anti-inflammatory effects that are different from what would usually be expected. It increases the circulation of IL-10 and IL-1ra and inhibits lipopolysaccharide (LPS)-induced TNF-α (32). Last, but not least, Stewart et al. showed that regular exercise over 12 weeks reduced the TLR4 expression in CD14^+^ cells, thereby contributing to a less active inflammatory environment (33).

**Figure 1:**
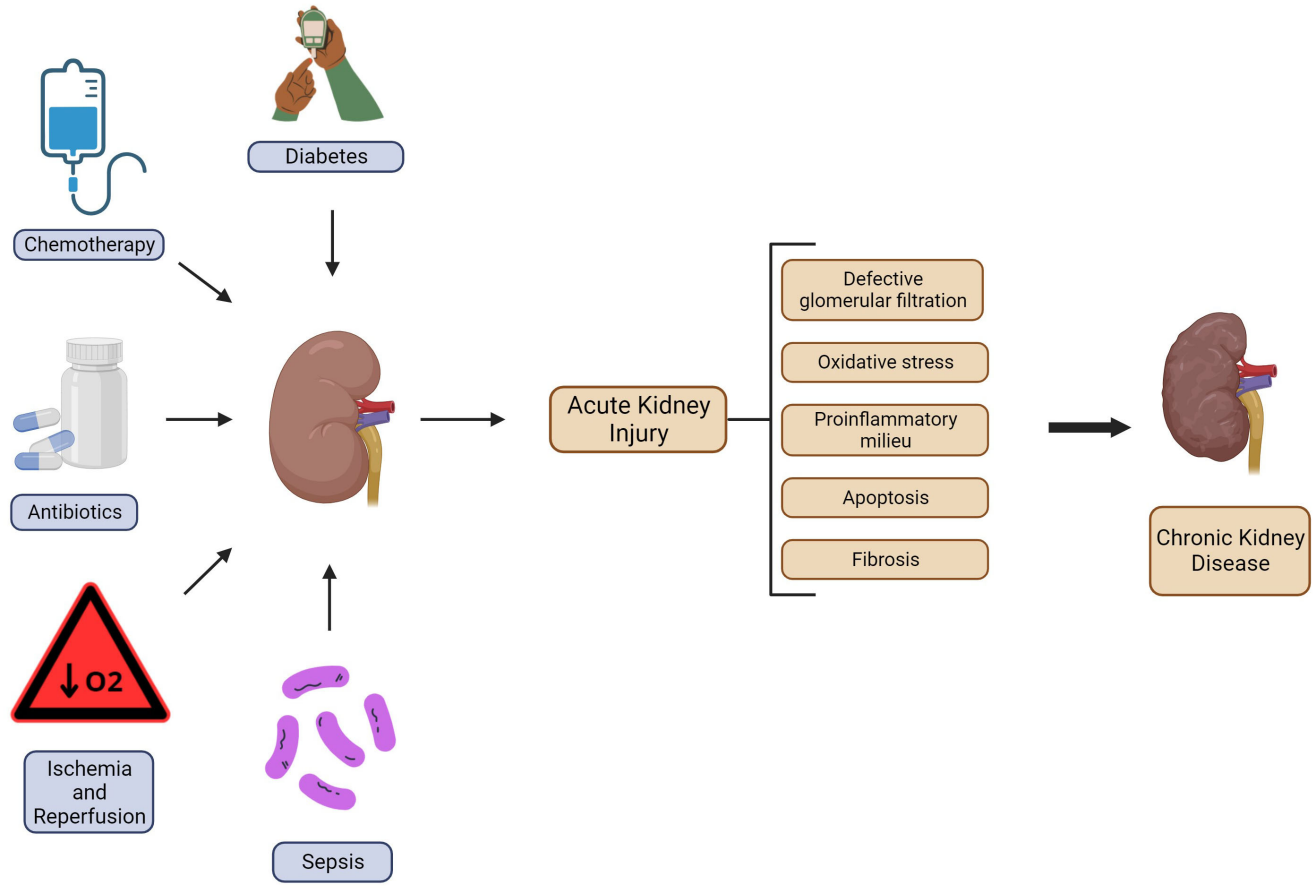
Schematic representation of AKI triggers and the mechanisms associated with CKD progression. Several situations can lead to AKI; the most common are diabetes, chemotherapy, use of antibiotics, ischemia and reperfusion, and sepsis. Once there is the onset of this pathologic condition, it can cause functional depletion, histologic damage, and a shift in the inflammatory response toward a proinflammatory milieu. In more severe cases it can progress to the development of CKD. Created with BioRender.com.

In this review, we have focused on the effects of regular aerobic exercise on immune system regulation and the therapeutic potential of physical exercise in treating AKI induced by different triggers (hypoperfusion/ischemia and reperfusion, sepsis, diabetes, antibiotic use, chemotherapy, etc.) as shown in **Figure 1**.

## Ischemia and reperfusion

Renal hypoperfusion contributes to AKI development (3) and is extensively studied because of its contribution to the understanding of ischemia and reperfusion (IR) during kidney transplantation. A systematic review evaluating kidney transplant recipients, with structured training protocols consisting of exercises three times a week for 3–6 months, showed improvements in cardiorespiratory fitness and muscle strength (34). It has also been shown that cardiovascular reserves are relevant survival predictors for CKD and kidney transplantation (35). Based on these data, we assembled some studies that sought the effects of exercise on renal function in different hypoperfusion conditions.

Kidney IR is a widely utilized model based on clamping either one or both kidneys for a short period of time, which comprises the ischemia, and then removing the clamps to allow reperfusion. This process is known to cause great local inflammation that can affect the engraftment (36). In the Lima et al. (2019) (37) study, before IR induction, Wistar rats were subjected to 4 weeks of training five times a week, with progressive intensity and duration, not achieving exhaustion, and this group was named exercise plus ischemic/reperfusion (EX+IR). EX+IR rats presented with lower levels of serum creatinine, blood urea nitrogen (BUN), and proteinuria after 48 h of reperfusion than sedentary rats. The EX+IR group also had less renal tissue injury, probably due to the controlled cell death observed by caspase-3 expression (37). Similar results were observed in the Lee et al. (2010) study, in which rats underwent exercise training (running for 30 min a day, five times a week, for 4 weeks), and were then subjected to hemorrhagic shock (EHS). In addition to having a lower increase in BUN and serum creatinine levels, rats that went through the exercise program had higher survival rates and lower levels of the inflammatory marker lactate dehydrogenase (LDH) than rats that did not (38). These results show that preconditioning regular aerobic exercise is efficient in attenuating AKI outcomes.

Another instability of hemodynamic hypoperfusion that can lead to AKI is myocardial infarction, known as the “cardio-renal syndrome” (6). A myocardial infarction experimental model was performed in Wistar rats with a permanent left anterior descending artery ligation. Four weeks after myocardial infarction, the rats were randomly distributed into exercise-based or sedentary-based intervention groups, with and without L-arginine supplementation (39). The exercise protocol consisted of running on a treadmill at a speed of 10–17 meters per minute, for 20–50 min a day for 10 weeks. L-arginine has been used as a precursor of nitric oxide (NO), which has physiological properties such as vasodilatation and the reduction of oxidative stress (40). No significant difference was found in serum creatinine measurements among groups, although BUN levels were lower in the exercise group, with no difference from those in the exercise group that received supplementation. After 10 weeks, training with and without L-arginine supplementation decreased lipid peroxidation in myocardial infarction rats, leading to less kidney oxidative stress. This indicates that exercise after cardio-renal syndrome could ameliorate kidney function by diminishing inflammatory innate immunity (39).

## Sepsis

Sepsis is a severe inflammatory disorder with high mortality and poor outcomes, causing end-organ dysfunction; it is a recurrent complication of AKI that raises mortality (6, 41). It has been shown that moderate and intense exercise can modulate neutrophils by improving the function of chemotaxis, phagocytosis, and microbicide (42).

In an animal sepsis model, after 4 weeks of treadmill training, rats received intravenously endotoxin (LPS from *Escherichia coli*). The training consisted of running on a treadmill at a speed of 12 meters per minute (m/min) for 15 min daily in the first week, 15 m/min for 30 min in the second week, 18m/min for 45 min in the third week, and 21 m/min for 60 min in the fourth week. In the acute phase, the trained group had increased neutrophils, and after 72 h the number of circulating lymphocytes was higher than in the sedentary group. The trained group also demonstrated attenuated septic responses and associated alterations due to endotoxin administration, and a reduced incidence of septic shock. They also exhibited less organ dysfunction measured by diminished levels of BUN and serum creatinine (kidney function biomarkers), creatine phosphokinase, LDH, aspartate aminotransferase, and alanine aminotransferase (liver damage biomarkers), and amylase and lipase (pancreas function biomarkers). The histopathological analysis also showed that endotoxin-induced organ damage was abrogated in trained rats. Exercise also attenuated the endotoxin-induced release of NO, free radicals, and proinflammatory cytokines such as TNF-α and IL-1β.

Mice subjected to the cecal ligation and puncture (CLP) sepsis model and that had previously trained on a treadmill at a speed of 8–12 m/min for 30 min three times a week for 8 weeks, showed less organ injury, fewer cell infiltration in the lungs, and increased survival rates. The trained animals presented with lower levels of the proinflammatory cytokines IL-1β, IL-6, and IL-8 in the peritoneal fluid and reduced IL-8 levels in the plasma. They also showed higher levels of the anti-inflammatory cytokine IL-10 in the peritoneal fluid and plasma, which demonstrates an attenuated inflammatory response (43).

In a severe polymicrobial sepsis model (fecal-induced peritonitis), mice were trained on a treadmill for 5 days a week for 6 weeks, with a stepwise adjustment of the duration and intensity of exercise from the first until the sixth week. Signs of morbidity were reduced and there was an increased survival rate (20% of the trained group survived while all the untrained animals died). In the acute phase post sepsis induction (6 h), trained mice demonstrated significantly lower concentrations of IL-6 and IL-10, with no difference in bacterial clearance between groups. After 24 hours IL-6 levels remained lower and increased bacterial clearance in all tissues was observed as being more significant in the lungs and blood. Moreover, regular exercise reduced signs of sepsis-associated AKI, with less tubular damage and twofold lower BUN levels (44). Trained mice also exhibited increased arginine and lysophosphatidylcholines plasma levels, metabolites that are likely to contribute to an immunomodulatory response that is beneficial for the host.

A major problem in sepsis is the phenomenon of a cytokine storm (45), which causes an amplified dysfunction response and depends on several factors, including individual genetics. Recent studies regarding COVID-19 sepsis and cytokine storms have helped to elucidate the relationship between this phenomenon and kidney injury, once the host immune response plays a major role in disease severity (46). Those studies have shown that cytokine-release syndrome causes tubular damage and endothelial dysfunction in kidney tissue, with IL-6 and IFNγ being the most relevant cytokines (47, 48). Those proinflammatory cytokines have their gene expression induced by the NF-kB pathway (47). In addition, IFNγ produced by natural killer cells in the tubulointerstitial tissue was demonstrated to be related to renal fibrosis and CKD progression (49). Furthermore, the systemic inflammation caused by a cytokine storm leads to septic shock, reducing cardiac output, and inducing pre-renal AKI (48). In addition, physical exercise in COVID-19 patients was shown to decrease inflammation, downregulating the mTOR and NF-kB pathways and possibly contributing to the prevention of severe outcomes (50). Thus, regular exercise has the potential to, at least partially, prevent cytokine storms by presenting a less exacerbated immune response against pathogen-associated molecular patterns (PAMPs) and DAMPs.

All of the abovementioned studies were conducted under aerobic exercise protocols, which are structured activities designed to increase cardiovascular and respiratory functions (10). Hence, aerobic physical exercise seems to protect against sepsis-associated organ injuries and reduce fatal outcomes and proinflammatory responses. These effects occur possibly due to the modulation of the immune system and the adaptation of kidney tissue, leading to a more efficient and balanced immune response. Other types of exercise, such as strength, mobility, and balance training, were not evaluated; therefore, further investigation is required to determine their contribution to sepsis-associated organ injuries.

## Diabetes

It is well known that diabetes mellitus (DM) is associated with important clinical complications such as nephropathy, which is the most harmful chronic complication and increases the risk of CKD development (51, 52). Regular exercise training is highly recommended to patients with chronic diseases such as DM or hypertension, because of the benefits in preventing and managing the disease and better control of the symptoms, which has an impact on the disease’s outcome (53).

It has been shown in rats that aerobic exercise performed prior to DM induction improved metabolic control by reducing weight and controlling glycemic levels. The exercise protocol consisted of running on a treadmill 5 days a week for 4 weeks, for 20 min in the first week and increasing up to 1h by the end of the fourth week. The exercise also ameliorated renal function by increasing the glomerular filtration rate and decreasing proteinuria. The aerobic training attenuated structural changes, with fewer tubular and glomerular injuries as measured by the tubulointerstitial lesions score and glomerulosclerosis index. The trained group also presented less expansion of the extracellular matrix, with a decreased expression of TGF-β, and attenuated the accumulation of fibronectin and collagen IV. The same result was maintained, or even improved, with continuous exercise for 8 weeks after DM induction (54).

Similar results have been seen in a mouse model of moderate aerobic exercise training for 6 weeks, with 1 h of running on a treadmill per day at a speed of 12 m/min, 6 days/week, and after diabetes-induced renal injury. The exercise group achieved better glycemic control and decreased proteinuria and collagen IV levels. Furthermore, it was observed that aerobic exercise training mitigates mitochondrial dysfunction, with decreased superoxide production and improved membrane potential and ATP production. Tang et al. also found that aerobic exercise increased the renal expression of the immune modulator complex Sirtuin (Sirt)1/Peroxisome proliferator-activated receptor-gamma coactivator (PGC)-1α and this was correlated to a protective effect on kidney injury (55).

Another study conducted 8 weeks of training after DM induction, in which rats were subjected to exercise training that consisted of 60 min per day of running on a treadmill at 16 m/min, 5 days a week. It showed that exercise could improve renal function and reduce levels of albuminuria, lipid peroxidation, and thiobarbituric acid-reactive substances. These results suggest a decreased level of oxidative stress, showing an ameliorated urine excretion of NO and an absence of renal lesions (56). NO is synthesized in the tubular endothelium, over other tissues, and it is responsible for hemodynamic regulation, tubular function, vascularization control, and redox balance by increasing antioxidant products (57, 58).

Overall, exercise was shown to have renoprotective effects, preventing renal dysfunction and structural changes through immune regulation in diabetic-induced AKI.

## Antibiotics

Gentamicin (Garamycin) is a widely used antibiotic in the treatment of severe Gram-negative bacterial infections. Unfortunately, it also causes a reduction in the glomerular filtration rate, increases the likelihood of proximal tubular necrosis, and induces oxidative stress in renal tissue, which is characterized by nephrotoxicity and usually leads to AKI (8, 59). Gentamicin is an aminoglycoside and the proximal tubule cells of the renal cortex have the ability to concentrate aminoglycosides several times more than plasma levels. Once gentamicin gets inside the cell it causes oxidative stress by acting on mitochondria and damaging cell membrane by phospholipidosis, leading to kidney injury (59, 60).

A study conducted in 2011 showed that rats treated with gentamicin for 10 days presented with decreased renal function and tissue injury, elevated NO levels in serum, diminished NO levels in urine, and no signs of expression of inducible nitric oxide synthase in the kidneys. All parameters were restored after 30 days without gentamicin (61). A similar study showed that 30 days of moderate running on a treadmill after 10 days of gentamicin treatment recovered NO urinary excretion. The rats ran at 16 m/min for 60 min a day, 5 days a week, for 30 days. The authors also evaluated another inflammatory component, ROS, which was measured through the presence of their byproduct, thiobarbituric acid-reactive substances. After the exercise, those substances were decreased in plasma, urine, and renal tissue, and there was increased production of the antioxidant defenses of the kidney catalase and glutathione. Gentamicin treatment elevated the pro-fibrotic cytokine TGF-β production and the lymphomononuclear infiltration in the renal tissue, which was restored to steady-state levels with exercise (62).

Taurine is an amino acid-like compound commonly found in mammalian tissues and is known to have a role in cellular plasma membrane stabilization and osmoregulation and have antioxidant effects (63). The use of taurine supplementation was shown to soothe kidney damage caused by gentamicin resulting in better renal function, more antioxidant agent activity, less hypoxia, and fewer histologic changes (64, 65). It has also been demonstrated that exercise increases taurine plasma concentrations (66–68) and decreases thiobarbituric acid-reactive substances (69). Because taurine is an essential amino acid found in various animal tissues in elevated concentrations, one could argue that the mechanism involved in gentamicin-nephrotoxicity reduction after exercise could be a result of the rise in taurine. Although further studies are required, these data suggest that moderate and regular exercise could improve the process of gentamicin-induced AKI recovery.

## Chemotherapy

Chemotherapy is one of the oldest forms of cancer treatment, and it remains the most used option. Exercise has been proposed as a therapy to ameliorate the physical and psychological side effects of chemotherapy. Nephrotoxicity is one notable side effect associated with the use of chemotherapies, such as cisplatin or doxorubicin. Unfortunately, there is no efficient therapy to reduce cisplatin-induced nephrotoxicity (42, 70, 71).

A study with rats consisted of 30 min of continuous running on a treadmill, 5 days a week for 4 weeks prior to an intraperitoneal injection of cisplatin (5 mg/kg) or saline, in comparison to a sedentary-cisplatin or control group. Previous training ameliorated renal function and resulted in fewer tubulointerstitial lesions, less inflammation (as evidenced by decreased macrophage infiltration), and diminished IL-1β, as well as decreased urinary excretion of MCP-1 and TGF-β. Increased NO levels in renal tissue and phosphorylation of endothelial NO synthase (eNOS) were observed, contributing to renoprotection, which possibly indicates a preserved endothelial function (72).

Similar results were described by our group using mice that underwent exercise training for 6 weeks running on a treadmill 5 days a week, with the time spent training each day increasing from 30 min to 60 min each week. Exercise attenuated renal dysfunction and inflammation, with reduced cell death and increased expressions of IL-6 and heme oxygenase (HO-1) in the kidneys (73). HO-1 is an enzyme that catalyzes heme degradation. It is known to be a regulator of the inflammatory response as an antioxidant agent and is associated with cytoprotection in AKI and other renal diseases (74, 75).

We also investigated the role of adaptive immune cells in physical exercise AKI protection. Corroborating our previous data, less damage and fewer inflammatory profiles were seen. The training mouse group (submitted to the same training protocol used for Miyagi et al. (2014)) showed less renal dysfunction (serum creatinine levels) and less tissue damage, as shown by decreased KIM-1 expressions. The study suggests a different modulation of T cells in the kidneys after exercise, showing fewer CD4^+^ T cells with less activation (CD4^+^CD25^+^ and CD4^+^CD69^+^ decreased populations), but also a decrease in regulatory T cells (Treg CD4^+^FOXP3), which is believed to be a reflex of less T helper recruitment in general. Similarly, IL-10 and TNF-α production by CD4^+^ T cells in kidney lymph nodes were restored to basal levels in the exercise group when compared with the cisplatin-sedentary group (76).

Cardoso et al. (77) demonstrated that rats that regularly exercised on a voluntary running wheel for 2 months after doxorubicin-induced AKI showed less damage and better tissue recovery. They had fewer renal injuries, inflammatory responses, and collagen deposition in tubules, and milder thickening of the Bowman’s capsule. Although normal restorations could not be verified, it is evident that the attenuation of kidney damage after exercise likely improves kidney function. These results suggest that aerobic exercise is able to result in a less pronounced inflammatory profile by producing a lower amount of cytokines, leading to decreased kidney injuries (76), and influencing both innate and adaptive immune responses.

Other interesting data are that paclitaxel, a chemotherapic, has been associated with the amelioration of sepsis-induced AKI (78). The use of this chemotherapic in low doses seems to diminish fibrosis and modify miRNA expression patterns in kidney disease models in rats (79, 80). In this context, low doses of paclitaxel effects and immune modulation may have a synergic action with aerobic exercise effects. It is also interesting that the deacetylation of p53 has alleviated sepsis-induced AKI in an animal model (81) and that p53 plays an important role in AKI pathogenesis and repair (82). These data support the idea that cancer and AKI may have an intimate relationship, inclusive in their treatments, that is not yet well studied.

Collectively, these studies indicate that regular exercise might diminish AKI through downregulation of innate and adaptive immune effector responses.

## Discussion

It is unquestionable that physical activity improves health, and it is positively correlated to the prevention and management of chronic diseases and the improvement of cardiologic and metabolic outcomes. It can also increase the expression of various growth factors and neurotransmitters in the brain, resulting in improvements in cognition, neurogenesis, and preventing different psychological diseases. Furthermore, exercise modulates the immune system, modifying its interaction with all tissues. Conversely, it is expected that a variety of responses among molecules, cell types, tissue, and organs stimulated during various types of exercise diverge in intensity, duration, and individual personal characteristics (83–85). AKI is an acute disease with the potential for late complications leading to CKD development with dialysis dependence (6), and it can affect transplant engraftment. The prevention and treatment of AKI still require major attention and further studies.

During exercise, our kidneys work to maintain adequate fluid and electrolyte homeostasis. In extenuating high-intensity exercise, blood redistribution to active body parts occurs, causing a decrease in renal perfusion and the filtration ratio (14, 86). On the other hand, moderate physical activity is also correlated with better kidney function and less kidney damage (87) and there are few solid studies describing the benefits of exercise in mitigating or even preventing AKI.

Tissue repair is mostly conducted by inflammation and fibrosis with collagen disposition. However, when this process occurs it may result in cell and tissue malfunction, and eventually culminate in CKD (88).

**Figure 2:**
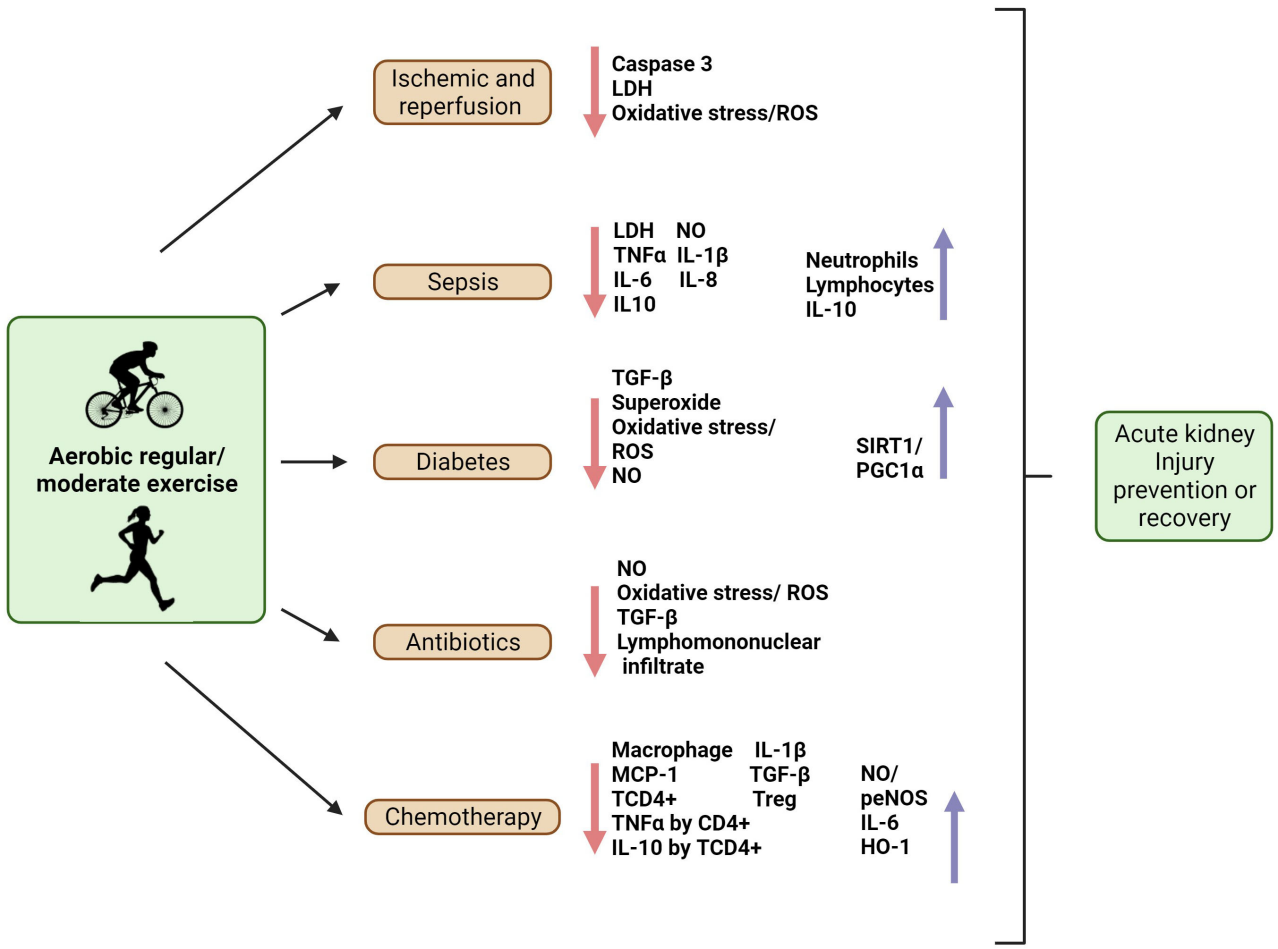
Aerobic regular/moderate exercise as an immunomodulator to prevent AKI or aid in the recovery of the kidney from AKI. In ischemia and reperfusion, exercise was shown to diminish caspase 3, oxidative stress (ROS), and LDH levels, providing a better recovery or preventing lesions in these situations. In the sepsis model, exercise promoted the recovery of neutrophils and lymphocytes, and decreased inflammatory markers such as LDH, NO, TNFα, and IL-1β, IL-6, and IL-8 levels; it might also modulate IL-10 levels. In diabetes, physical exercise prevents kidney injury by decreasing TGF-β, superoxide, oxidative stress, ROS, NO, and increasing Sirtuin (SIRT)1/Peroxisome proliferator-activated receptor-gamma coactivator (PGC)-1α. Exercise can prevent AKI induced by antibiotics by decreasing NO, ROS, TGF- β, and leukocyte infiltration. In chemotherapy-induced AKI, exercise can prevent kidney injury by decreasing macrophage, monocyte chemoattractant protein-1 (MCP-1), IL-1β, TGF-β, CD4^+^ T cells (TCD4^+^), regulatory T cells (Treg), TNF-α, and IL-10 levels produced by TCD4^+^, and inducing the production of NO, phosphorylation of endothelial NO synthase (p-eNOS), heme oxygenase (HO-)1, and IL-6. Created with BioRender.com.

Moderate exercise has a positive impact on the immune system (42, 89). Physical exercise was able to modulate immune cells to a regulatory phenotype, associated with an increase in anti-inflammatory cytokines, regulating cytokine balance, such as TNF-α and IL-10 levels, as well as increases in IL-6 levels, and decreases of reactive oxygen and nitrogen species. This environment is less conducive to apoptosis and fibrotic persistence, therefore preserving glomerular filtration, preventing AKI, and diminishing the risk of CKD (4), as shown in the schematic in **Figure 2**.

These findings support the prescription of moderate and regular aerobic exercise for patients who are at risk of developing AKI and also for patients who have already been diagnosed with AKI. As physical exercise is a simple and low-cost intervention, this could be broadly applied at all levels of the health care system, producing great results by improving patients’ health and diminishing the burden of the disease.

## Conclusion

In conclusion, regular practice of moderate physical exercise has an impact on the immune system, favoring a regulatory and anti-inflammatory profile, which prevents the development of and aids recovery from AKI. Protection from AKI induced by several triggers minimizes the risk of CKD. Under supervision, physical exercise has great potential for preventing AKI or treating patients with AKI.

## Author contributions

AC-N and LB-A have contributed equally to this study. LB-A, WP, NC, and MA designed the review. AC-N, LB-A, EB-J, and MA wrote the text. AC-N, WP, NC, and MA revised the text and added intellectual content. AC-N, LB-A, EB-J, WP, and MA proofread the review. MA is the corresponding author. All authors contributed to the article and approved the submitted version.
